# Tricyclic antidepressants and the incidence of certain cancers: a study using the GPRD

**DOI:** 10.1038/sj.bjc.6605996

**Published:** 2010-11-16

**Authors:** A J Walker, T Card, T E Bates, K Muir

**Affiliations:** 1 Division of Epidemiology and Public Health, School of Community Health Sciences, University of Nottingham, Nottingham NG7 2UH, UK; 2 Department of Gastroenterology, King's Mill Hospital, Mansfield Road, Sutton-in-Ashfield, Nottinghamshire, NG17 4JL, UK; 3 Nottingham Digestive Diseases Centre, NIHR Biomedical Research Unit, University of Nottingham, Nottingham NG7 2UH, UK; 4 School of Natural and Applied Sciences, University of Lincoln, Brayford Pool, Lincoln LN6 7TS, UK; 5 New-Use Therapeutics Limited, BioCity Nottingham, Pennyfoot Street, Nottingham NG1 1GF, UK; 6 Health Sciences Research Institute, Warwick Medical School, Warwick University, Coventry CV4 7AL, UK

**Keywords:** tricyclic antidepressants, glioma, colorectal cancer, GPRD

## Abstract

**Background:**

Several studies suggest links between cancer and tricyclic antidepressant use.

**Methods:**

A case–control study using the General Practice Research Database examined whether previous tricyclic usage was associated with reduced incidence of brain (with glioma as a sub-category), breast, colorectal, lung and prostate cancers. Conditional logistic regression adjusted for age, gender, general practice, depression, smoking, body mass index, alcohol use and non-steroidal anti-inflammatory drug use.

**Results:**

A total of 31 953 cancers were identified, each matched with up to two controls. We found a statistically significant reduction in tricyclic prescriptions compared with controls in glioma (odds ratio (OR) =0.59, 95% confidence interval (CI)=0.42–0.81) and colorectal cancer patients (OR=0.84, CI=0.75–0.94). These effects were dose-dependent (*P*-values for trend, glioma=0.0005, colorectal=0.001) and time-dependant (*P*-values for trend glioma=0.0005, colorectal=0.0086). The effects were cancer-type specific, with lung, breast and prostate cancers largely unaffected by antidepressant use.

**Conclusion:**

The biologically plausible, specific and dose- and time-dependant inverse association that we have found suggests that tricyclics may have potential for prevention of both colorectal cancer and glioma.

Prevention of disease is better than its cure. In the case of cancer, where complete cure is often not possible, the strategies available for cancer prevention are still limited. However, the vast numbers of existing drugs used daily by millions of people are a potential source of agents that may help to prevent or even treat cancer. Despite notable examples of success such as the non-steroidal anti-inflammatory drugs (NSAIDs) (Cuzick *et al*, 2009) and statins (Kaye and Jick, 2004), few such agents have however yet been discovered.

Tricyclic antidepressants (TCAs) are widely prescribed for a variety of conditions, including depression, anxiety and insomnia. Although tricyclics have received some attention in epidemiological studies as possible carcinogens owing to their putative genotoxic activity (van Schaik and Graf, 1991), these have been inconclusive (Fulton-Kehoe *et al*, 2006; Xu *et al*, 2006; Tamim *et al*, 2007; Toh *et al*, 2007). There is substantial evidence that conflicts with this carcinogenic view of tricyclics, as particularly chlorimipramine (clomipramine), although also imipramine, citalopram, amitriptyline and desipramine, have shown anticancer effects (Xia *et al*, 1999; Arimochi and Morita, 2006). Tricyclics have shown cytotoxic actions in various cancer cell lines including glioma cells (Xia *et al*, 1999; Daley *et al*, 2005; Levkovitz *et al*, 2005) and colorectal cancer cells (Arimochi and Morita, 2006), perhaps attributable to inhibition of mitochondrial complex III activity, leading to a decrease in mitochondrial membrane potential, and apoptosis (Weinbach *et al*, 1986; Daley *et al*, 2005). Animal studies substantiate an anticancer action in overcoming drug resistance in various cancer models, such as sarcoma, lymphocytic leukaemia and leukaemia grown as a solid tumour (Tsuruo *et al*, 1983; Merry *et al*, 1991; Pommerenke and Volm, 1995). Much attention has focussed on glioma as a target for tricyclics and there have been some preliminary clinical studies in humans using chlorimipramine therapeutically (Beaney *et al*, 2005).

Based upon their apparent sensitivity to tricyclics *in vitro,* we hypothesised that they would have a protective effect against glioma and colorectal cancer. We therefore conducted a case–control study using the GPRD to investigate this. In addition to examining these two tumour types, we also studied certain other cancers to look for specificity of any effect.

## Materials and Methods

A matched case–control study was used to investigate possible relations between drug usage and cancer incidence. Cases were defined as any person with a recorded diagnosis of brain tumour, breast, colorectal, lung or prostate cancer within the GPRD (diagnosis codes available on request); gliomas were considered separately. We excluded cases with <5 years of follow-up before the first recorded diagnosis of the relevant cancer, aged <18 years or with a diagnosis of any malignancy before the index cancer. Controls were individuals contributing data at the time of the case index date, with at least 5 years of follow-up before that date and with no recorded diagnosis of cancer. They were matched to cases by year of birth, gender and GP practice in a ratio of 2 : 1 where possible.

The GPRD is a prospectively gathered, anonymised database encompassing around 500 GP practices throughout the United Kingdom, and is the largest of its type in the world, with around 45 million patient-years of data spread across ∼6.5 million patients. It provides data on patients including clinical diagnoses, treatments and outcomes. The database was established in 1987, with its development corresponding to the increased computerisation of GP practices, and its validity has been well documented (Jick *et al*, 1991, 2003; Fombonne *et al*, 2004; Herrett *et al*, 2010).

The primary exposure was the use of any tricyclic antidepressant (section 4.3.3 of the British National Formulary (BNF)). We abstracted data on all such prescriptions at least 1 year before the date of diagnosis of the index cancer (or the equivalent pseudo-diagnosis date of the cancer for controls). We first created a binary variable defining a tricyclic user as anyone with a repeat prescription for any tricyclic antidepressant, and comparing these with non-users. To allow assessment of dose response we determined the mean dose received across all exposed days for each patient. This was standardised across drug types by dividing by the maximum recommended doses for each drug (determined from the BNF). Each patient was then placed in a ‘high dose’, ‘low dose’ or ‘unexposed’ category, such that those exposed were divided equally into high- and low-dose categories. Length/consistency of exposure was assessed by determining the number of days of exposure over a 10-year period before the index date. Any patient contributing data for less time was excluded for this part of the analysis. Exposed patients were then divided into two groups of equal size. In order to assess any potential confounding by indication, SSRI use was investigated by categorising those exposed to only SSRIs, only tricyclics, and both SSRIs and tricyclics. We extracted data on smoking status, body mass index (BMI), alcohol use, diagnosis of depression and prescriptions for NSAIDs and statins, which we considered as possible confounders.

### Statistical methods

Data were analysed with conditional logistic regression, initially using univariate analysis, and then using a multivariate model. Results were presented as odds ratios (ORs), with 95% confidence intervals (CIs). Potential confounders were retained in the model if their inclusion produced a 10% variation in the measured size of effect. Analyses were performed on all cancer types together, followed by individual cancer types to look for specific effects. Trend was tested for by including dose/exposure duration in the conditional logistic regression model as a single, ordered categorical variable, with *P*-values obtained using the likelihood-ratio test. All data handling and analysis was done using Stata v10.1 SE (Statacorp, College Station, TX, USA).

## Results

We identified 31 953 cases with which 61 591 controls were matched by age and gender. The cases consisted of 1372 cancers of the nervous system (of which 773 were gliomas), 10 293 of the breast, 6232 colorectal, 6537 of the lung and 6537 of the prostate. Median age of patients across all cancer types was 68.2. Females made up 50.7% (16 212) of the study and had a median age of 65.6, and males had a median age of 70.9. In all, 18.9% of cases and 17.6% of controls were exposed to one or more prescriptions for a tricyclic before one year before the index date. These data are summarised in Table 1.

As can be seen from Table 2, smoking was associated with an increased risk of cancer (OR=1.47, CI=1.42–1.53), almost entirely attributable to lung cancer, with an OR of 7.4 (CI=6.74–8.12) in smokers compared with non-smokers. There is a slight increase in risk of cancer for alcohol users (OR=1.09, CI=1.05–1.14), which was mostly because of breast (OR=1.11, CI=1.04–1.18) and colorectal cancers (OR=1.12, CI=1.02–1.23). There is an apparent decrease in cancer risk as BMI increases, mainly found in lung cancer patients, where the decrease is caused by confounding by smoking status, and is not statistically significant if only non-smokers are considered. NSAID use is not significantly different for all cancers together, but shows a significant reduction in colorectal cancer (OR=0.93, CI=0.87–0.99), as reported previously (Cuzick *et al*, 2009).

Analysis with tricyclic use coded as a binary variable (Table 3) demonstrates a significant reduction in tricyclic usage in colorectal cancer patients compared with controls (multivariate OR=0.84, CI=0.75–0.94). Tricyclic use is also significantly lower in glioma patients compared with controls, with a larger effect estimate (OR=0.59, CI=0.42–0.81). For most cancers, we found little evidence of confounding by the available potential confounders, except for lung cancer, where smoking status had a large confounding effect. The significant increase in tricyclic use observed in the univariate model (OR=1.30, CI=1.19–1.42) is greatly reduced when adjusted for confounders (OR=1.14, CI=1.02–1.28). Other cancers show little variation in tricyclic usage, with ORs very close to one.

For glioma, selective serotonin reuptake inhibitor (SSRI) use without tricyclic use showed little deviation between cases and controls (OR=0.96, CI=0.61–1.53). This is in contrast to tricyclic use with (OR=0.50, CI=0.27–0.92) and without (OR=0.74, CI=0.52–1.06) SSRI use. Colorectal cancer was similar to glioma in terms of exclusive SSRI use (OR=0.95, CI=0.81–1.12), and showed a similar pattern to the above binary analysis for tricyclic use with SSRI use (OR=0.85, CI=0.70–1.02) and exclusive tricyclic use (OR=0.85, CI=0.76–0.95).

When divided into ‘low’ and ‘high’ dose exposure (Table 4), tricyclic use is significantly lower at high doses for both glioma (OR=0.49, CI=0.30–0.78) and colorectal cancer (OR=0.79, CI=0.67–0.93). Highly significant trends validate these findings further for glioma (*P*=0.0005) and colorectal cancer (*P*=0.0010). Other cancer types were considered in the same way, although no notable or statistically significant trends were present (data not shown). We then investigated relationships with duration of tricyclic exposure. As with the dose analysis, long-term use is significantly lower in glioma (OR=0.36, CI=0.19–0.69) and colorectal cancer (OR=0.82, CI=0.68–0.97). Highly significant trends were observed again for glioma (*P*=0.0005) and colorectal cancer (*P*=0.0086). No notable or statistically significant trends were present for other cancers (data not shown).

## Discussion

This study finds that tricyclic use may be associated with a subsequent reduction in the risk of glioma and colorectal cancer. These protective effects appear to be specific to these particular cancers, as it was not observed for the other cancers studied, although we cannot rule out a protective effect in cancers not covered. The data also indicate that these apparent protective effects are greatest for patients receiving high-dose prescriptions over a long period.

Our data have certain important strengths. Our use of routinely collected general practice records (from the GPRD) ensured that there was no opportunity for recall bias to effect the ascertainment of exposures. In addition, by selecting all relevant malignancies within the population and a random sample of the suitable controls, we eliminated the possibility of selection bias. However, our data selection does have some weaknesses. Although the numerous validation studies of various diagnoses suggest that our outcomes were accurately coded, as the electronic recording of prescription data does for our primary exposure, we cannot be equally confident about the recording of all potential confounders. As Table 2 shows, there are much missing data with respect to smoking, obesity and alcohol, and therefore a potential for residual confounding by these factors. However, we believe that except for lung cancer where this is clearly an issue (and residual confounding by smoking might account for the positive association with tricyclics), the near total lack of confounding detected argues that any such residual will be minor. A potentially greater issue is that we lack any data on other potential confounders such as diet and exercise, and hence their impact on the results cannot be assessed.

Another strength of our study is that by studying several cancers, we have demonstrated that the protective effect of tricyclics appears to be specific to certain malignancies. Because of the study size, we have also been able to demonstrate that longer-term use and higher doses of tricyclics appear to give greater protection from glioma and colorectal cancers. As the proposed anticancer mechanism of action is a mitochondrial one (Daley *et al*, 2005) and independent of the psychoactive mechanism of action, there is reason to believe that these findings are generalisable, and not restricted only to a ‘depressed’ population.

Confounding by indication is important and if SSRIs are considered as were tricyclics, their pattern is similar (data not shown), possibly because their use is predictive of tricyclic use. If patients using SSRIs exclusively (i.e., no tricyclics) are considered, most of the effect disappears. The multivariate results were adjusted for diagnosis of depression, and this adjustment increased the apparent protective effect of tricyclics. It would seem more biologically plausible for depression to increase cancer risk than decrease it, and it is therefore likely that depression is a proxy for high-dose tricyclics (as it is usually treated with a higher dose than other indications, such as pain). This is supported by the relation with dose (where those without depression have a higher proportion of low dose and *vice versa*).

Previous studies have examined tricyclics and the incidence of colorectal (Xu *et al*, 2006), prostate (Tamim *et al*, 2007), breast (Cotterchio *et al*, 2000; Gonzalez-Perez and Garcia Rodriguez, 2005; Fulton-Kehoe *et al*, 2006; Wernli *et al*, 2009) and lung cancers (Toh *et al*, 2007), but have shown little consistency, and significant evidence has been found to link tricyclics with cancer. A study on colorectal cancer (Xu *et al*, 2006) hypothesised that tricyclics are genotoxic, and therefore increase cancer risk, but, their results suggest a nonsignificant protective effect, as did another recent study (Patricia *et al*, 2009). Our findings for colorectal cancer fit well with these data, the statistically significant protection being a function of our greater numbers. The previous lung cancer study (Toh *et al*, 2007) showed an apparent increase in risk with tricyclic use, which is largely mitigated by adjusting for confounders (including smoking status), as with our data.

We have found a significant reduction in incidence of colorectal cancer and glioma, consistent with previous laboratory evidence (Daley *et al*, 2005), and not inconsistent with other epidemiological studies. The findings show specificity of protection for those malignancies originally hypothesised and show a dose-response and a clear temporal relationship. It is credible that our associations may be causal, although the modest size of the effect limits the potential of these drugs as a chemopreventative agent in the general population. As glioma is a rare cancer with ill-defined high-risk groups, prescribing chemopreventative drugs are of limited value in the general population; thus, we estimate that ∼60 000 people would need to be treated (for >117 days) in order to prevent one glioma. Groups at increased risk of colorectal cancer (e.g., those with a familial or other genetic predisposition) might still represent an appropriate group for an RCT as they would have the best chance of benefiting from chemoprevention, here although one would need to balance potential benefits against possible side effects.

If the antineoplastic effects of tricyclics are to be therapeutically useful, it is likely to follow identification of a potent compound within the group, or in postdiagnosis treatment of colorectal cancer and glioma. Thus, aspirin, a recognised prophylactic for colorectal cancer, has recently been found to reduce colorectal cancer mortality when used after diagnosis (Chan *et al*, 2009; Zell *et al*, 2009), and to an extent achieved previously only by far more toxic compounds. It would therefore be useful to determine whether tricyclics have similar effects on colorectal cancer and glioma.

## Figures and Tables

**Table 1:** Characteristics

	**Cases**		**Controls**	
**Cancer**	**Number**	**%**	**Number**	**%**
*All*				
Total	31 953		61 591	
Male	15 740	49.3	29 998	48.7
Female	16 212	50.7	31 593	51.3
Mean age		68.3		
				
*Glioma*				
Total	773		1502	
Male	468	60.3	906	60.5
Female	305	39.7	596	39.5
Mean age		60.1		
				
*Colorectal*				
Total	6232		12 010	
Male	3496	56.1	6704	55.8
Female	2736	43.9	5306	44.2
Mean age		70.9		
				
*Brain (excl glioma)*				
Total	599		1164	
Male	214	35.7	413	35.5
Female	385	64.3	751	64.5
Mean age		65.8		
				
*Breast*				
Total	10 293		20 096	
Male	—	—	—	—
Female	10 293	100.0	20 096	100.0
Mean age		62.5		
				
*Lung*				
Total	6537		12 514	
Male	4035	61.73	7653	61.16
Female	2502	38.27	4861	38.84
Mean age		71.0		
				
*Prostate*				
Total	7531		14 329	
Male	7531	100.0	14 329	100.0
Female	—	—	—	—
Mean age		72.5		

A summary of the characteristics of the population, including for each of the cancers.

**Table 2:** Covariates

**Exposure**	**Status**	**Case**	**Control**	**OR**	**95%**	**CI**
Smoking status	No	15 369	32 153	1		
	Ex	5911	10 263	1.23	1.19	1.28
	Yes	7978	11 615	1.47	1.42	1.53
	Missing	2695	7560	0.69	0.65	0.72
Alcohol use	No	4778	9542	1		
	Ex	348	572	1.24	1.08	1.43
	Yes	21 028	38 670	1.09	1.05	1.14
	Missing	5799	12 807	0.87	0.83	0.91
Mean BMI	Normal	10 713	19 466	1		
	Underweight	701	1020	1.26	1.14	1.39
	Overweight	10 086	19 005	0.96	0.93	1.00
	Obese	3191	6240	0.93	0.89	0.98
	Morbidly obese	961	2008	0.88	0.81	0.95
	Missing	6301	13 852	0.79	0.76	0.82
NSAID use	No	21 122	41 006	1		
	Yes	10 831	20 585	1.02	0.99	1.05
Statin use	No	26 957	51 933	1		
	Yes	4996	9658	1.00	0.96	1.04
Depression	No	23 890	47 458	1		
	Yes	8063	14 133	1.15	1.11	1.19

Abbreviations: Ex=former smoker; NSAID=non-steroidal anti-inflammatory drug; BMI=body mass index; OR=odds ratio; CI=confidence interval.

Changes in cancer risk for each of the covariates. These results are for all the studied cancer types grouped together, and hence do not describe differences in exposures between cancer type (see text).

**Table 3:** Binary analysis

				**Univariate**			**Multivariate** ^*^		
**Cancer type**	**Exposed**	**Case**	**Control**	**OR**	**95%**	**CI**	**OR**	**95%**	**CI**
Glioma	No	706	1317	1			1		
	Yes	67	185	0.66	0.49	0.89	0.59	0.42	0.81
Colorectal	No	5574	10 543	1			1		
	Yes	658	1467	0.85	0.77	0.94	0.84	0.75	0.94
Brain	No	505	1013	1			1		
(excl glioma)	Yes	94	151	1.26	0.95	1.67	1.00	0.72	1.38
Breast	No	8651	16 834	1			1		
	Yes	1642	3262	0.98	0.92	1.05	0.97	0.91	1.04
Lung	No	5555	10 992	1			1		
	Yes	982	1522	1.30	1.19	1.42	1.14	1.02	1.28
Prostate	No	6861	13 112	1			1		
	Yes	670	1217	1.06	0.96	1.17	0.94	0.84	1.04
All	No	27 841	53 790	1			1		
	Yes	4112	7801	1.03	0.99	1.07	0.93	0.89	0.97

Abbreviations: OR=odds ratio; CI=confidence interval. Patients with repeat prescriptions for tricyclics were defined as exposed. ^*^Corrected for smoking status, diagnosis of depression, alcohol use and BMI. NSAID use corrected for in colorectal cancer only.

**Table 4:** Dose and duration

**Cancer**	**Exposure status**	**Case**	**Control**	**OR**	**95%**	**CI**	***P*-trend**
*Dose*							
Glioma	Unexposed	707	1323	1			
	Low dose	38	97	0.67	0.45	1.01	
	High dose	28	82	0.49	0.30	0.78	0.0005
Colorectal	Unexposed	5592	10 595	1			
	Low	382	821	0.87	0.76	1.00	
	High	258	594	0.79	0.67	0.93	0.0010
							
*Duration (days)*							
Glioma	Unexposed	399	1051	1			
	1–117	22	71	0.65	0.37	1.13	
	>117	14	75	0.36	0.19	0.69	0.0005
Colorectal	Unexposed	3598	7752	1			
	1–117	345	747	0.84	0.70	1.01	
	>117	305	785	0.82	0.68	0.97	0.0086

Abbreviations: OR=odds ratio; CI=confidence interval. Glioma and colorectal cancer dose-response and duration results. Other cancer types did not show consistent dose-response relationships. All analyses in this table are corrected for smoking status, diagnosis of depression, alcohol use and BMI. NSAID use corrected for in colorectal cancer only.
